# Knowledge, Attitude, and Behavior of Medical Students from a Saudi University Toward Postgraduate Training

**DOI:** 10.7759/cureus.6356

**Published:** 2019-12-11

**Authors:** Ibrahim A Bin Ahmed, Sulaiman A Alrajeh, Abdulaziz A Alrajeh, Abdullah S Aldughaither, Abdullah A Kilani, Yazeed M Almotairy, Abdulrahman S Mirza, Abdullah A Alzaaqi, Abdulhameed S Qashqary

**Affiliations:** 1 Family Medicine, College of Medicine, Al-Imam Mohammad Ibn Saud Islamic University, Riyadh, SAU; 2 Medicine, College of Medicine, Al-Imam Mohammad Ibn Saud Islamic University, Riyadh, SAU; 3 Medicine, College of Medicine, Alfaisal University, Riyadh, SAU

**Keywords:** knowledge, attitude, behaviour, medical students, postgraduate training

## Abstract

Introduction

Choosing a postgraduate career path is a significant and complex decision for medical students. It involves gaining knowledge about a wide array of specialties to gain a comprehensive understanding of the specialties. The current study explored Imam Muhammad ibn Saud Islamic University, College of Medicine students’ perceived knowledge, attitudes, and behaviors toward postgraduate training

Methods

This was a cross-sectional, questionnaire-based survey study conducted in April 2016 to assess the knowledge, attitudes, and behaviors toward postgraduate training among the students of Imam Muhammad ibn Saud Islamic University, College of Medicine.

Results

Ninety-two students participated in the study. Of these, 74% were in the pre-clinical years (years 1, 2, and 3). The mean age of participants was 21. Students reported having a poor level of knowledge regarding the types of material covered in license exams (46.2% had responses classified under ‘poor’). Forty-six percent of students had a positive view of whether problem-based learning (PBL) prepared students for clinical scenarios. Of the total respondents, 24% reported having chosen a specialty while 78% of the students reported participating in extracurricular activities. Negative responses progressively decreased with each academic year.

Conclusion

This study highlights the significant lack of knowledge of medical students about the covered types of material in Saudi medical licensing exams. Nevertheless, they have adequate levels of awareness and acknowledgment, improve themselves, and modify their own weaknesses. Moreover, medical students show a positive attitude towards PBL that gives the ability to connect and relate to undergraduate studies and apply it to clinical practice. The delay in perceiving their own interests leads to an inadequate shift of focus. However, more studies are mandatory to explore the reasons behind the medical students’ lack of knowledge and the factors involved in choosing their specialties.

## Introduction

Medical education has undergone significant changes in recent years. More institutions are implementing competency-based curricula [1-4] with further emphasis being put on student-centered learning [5-8]. These changes prompt extensive exploration of students’ opinions of themselves as medical trainees as well as their outlook on their future training and practice. The College of Medicine at Imam Muhammad ibn Saud Islamic University employs a curriculum based on the aforementioned principles. The current study sought to explore students’ perceived knowledge, attitudes, and behaviors toward postgraduate training. We investigated students’ self-reported responses to medical training, residencies, and future practice.

## Materials and methods

Study design and participants

A cross-sectional, questionnaire-based survey study on male medical students, from the first to fifth years in Imam Mohammad Ibn Saud Islamic University in Riyadh, Saudi Arabia, conducted in April 2016. Data for the sample were collected through an online, self-administered questionnaire designed by using Google Forms. Preparatory year students were not included in the study, as they are not considered medical students yet (those students are conditionally accepted and become part of the college upon completion of the preparatory year and meeting college requirements). Interns were not part of the study, as they are considered graduates and not students of the college.

Questionnaire

Following a thorough review of the literature, we developed a questionnaire consisting of four sections with a total of 23 items. The first section contained four questions about demographic data. The second section, assessing knowledge, was made of five items. The third section focused on attitude and consisted of seven items. The last section assessed behavior via seven items. A pretest was made to ensure clarity and readability of the questionnaire.

Three types of questions were in the questionnaire, Likert scale-type, multiple-choice questions, and checkbox questions. Likert scale-type questions (with a scale of 1 to 5) were used to assess knowledge and attitude. Multiple-choice questions were used mainly to determine student behavior. Checkbox questions were implemented where a large scope of responses was expected.

The questionnaire was disseminated to participants via social media over a duration of 18 days.

Data analysis

IBM’s Statistical Package for Social Sciences 22 (IBM Corp, Armonk, NY) was utilized for data analysis. Likert-type scale questions were converted into categorical variables. Data were then compared and correlated via cross-tabulation and Pearson’s correlation. Responses of ‘very good’ and ‘good’ were categorized as ‘good,’ and, similarly, responses of ‘strongly agree’ and ‘agree’ were categorized as ‘agreed.’ Responses or ‘very poor’ and ‘poor’ were categorized as ‘poor,’ and responses of ‘strongly disagree’ and ‘disagree’ were categorized as ‘disagree.’ A two-sided p-value of 0.05 was considered significant while a p-value of 0.01 was considered highly significant.

## Results

Ninety-two students participated in the study. Seventy-four percent of students were in the pre-clinical years (years 1, 2, and 3). The mean age of participants was 21.93 ± 2.170. Thirty-two percent of participants had at least one first degree relative in the medical field. Sixty-seven percent of respondents reported having applied to medical school immediately after graduating high school. The demographic characteristics of participants are presented in Table 1.

**Table 1:** Demographic data

	Response		%
Academic year		1^st^ year	25.30%
		2^nd^ year	35.20%
		3^rd^ year	13.20%
		4^th^ year	9.80%
		5^th^ year	16.50%
Relatives in the medical field		Both parents	3.30%
		One parent	12%
		Brother/sister	16.30%
		Other	14.10%
		None	54.30%
Time between high school graduation and enrollment in medical school		Immediate	67.40%
		One year	27.20%
		Two or more years	5.40%

Knowledge

Students reported having a poor level of knowledge regarding the types of material covered in license exams (46.2% had responses classified under ‘poor’). However, they also reported having a good level of knowledge of their own weaknesses. The summary of the responses regarding knowledge is given in Table 2. A highly significant positive correlation was also found between students’ self-reported knowledge of different specialties and their self-reported knowledge of the application process for residency training (p< 0.001). There was also a significant correlation between participants' perceived knowledge of their own weaknesses and their view on whether license exams provide an accurate assessment of students (p=0.009).

**Table 2:** Responses in the knowledge section

	Response	%
Knowledge of application process for residency training	Good	29.7%
	Neutral	38.8%
	Poor	31.5%
Knowledge of types of materials covered in license exams	Good	23.1%
	Neutral	30.7%
	Poor	46.2%
Knowledge of students’ own weaknesses	Good	45.0%
	Neutral	27.5%
	Poor	27.5%

Attitude

Forty-six percent of students had a positive view of whether PBL prepared students for clinical scenarios. A significant correlation was found between students’ views of how undergraduate studies relate to future practice and their attitude toward PBL sessions (p=0.002). In response to a question about if medical schools prepare students for license exams, 46% of respondents disagreed while only 11% agreed. With high significance, students’ academic year was found to be inversely correlated to their opinion on whether medical schools prepare students for license exams (p=0.001). When given several options that describe different opinions on residency applications, 73% of students chose the option stating that available seats for residency training are not enough. A summary of the responses to attitude is given in Table 3.

**Table 3:** Responses in the attitude section PBL: problem-based learning

	Responses	%
Do you think available seats for residency training are enough	Yes	26.4%
	No	73.6%
Does medical school prepare students for license exams	Agree	11.0%
	Neutral	42.9%
	Disagree	46.1%
Does PBL prepare students for clinical scenario	Agree	46.2%
	Neutral	27.5%
	Disagree	26.3%
Do you think students in your batch pay attention to prepare for postgraduate training	Most do	26.6%
	Neutral	38.9%
	Most do not	34.5%

Behavior

**Figure 1:**
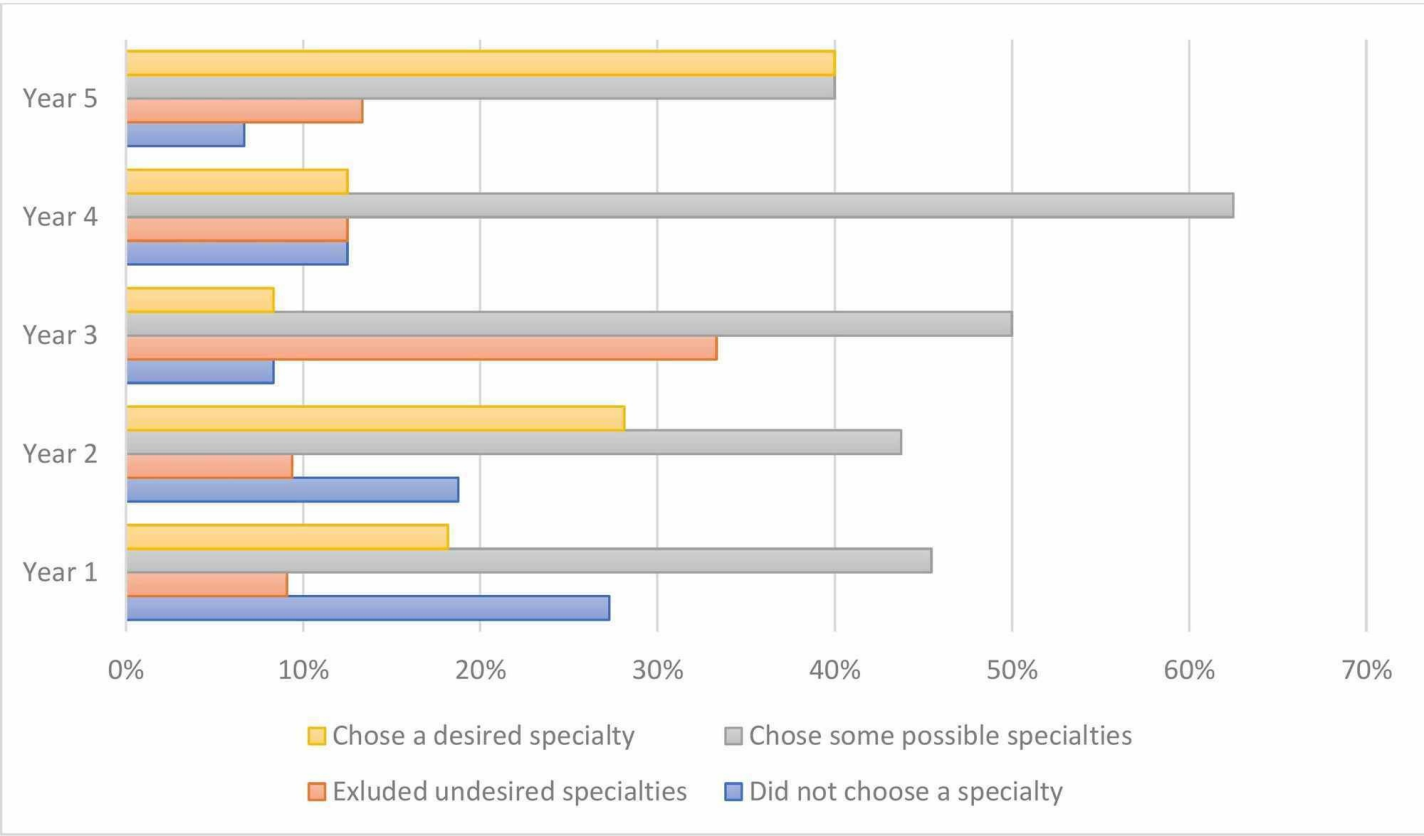
Comparison of specialty-choosing decisions among different academic years of medical students at Imam Muhammad Ibn Saud Islamic University in 2016

**Figure 2:**
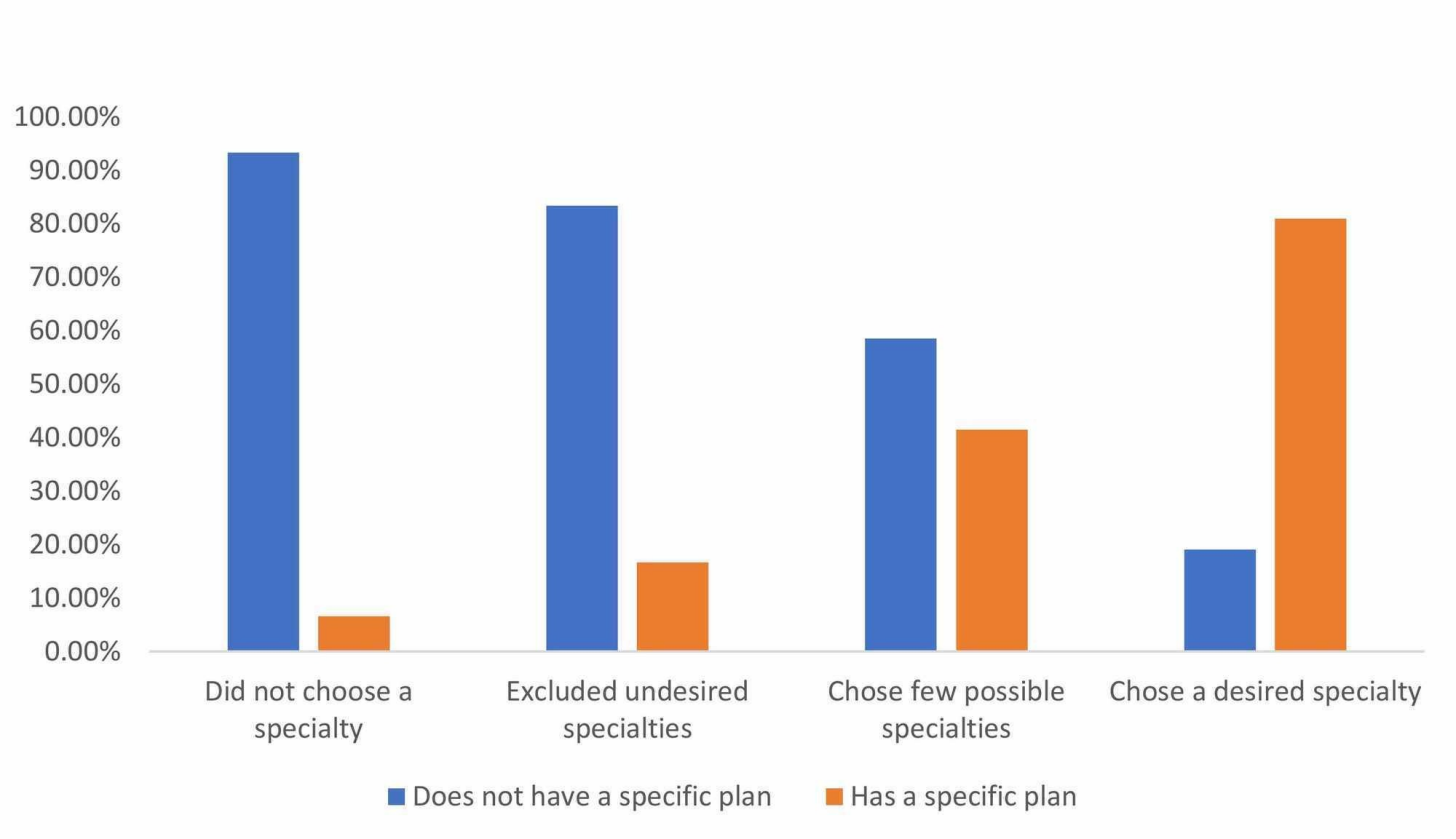
Responses regarding having plans to reach desired specialties as compared with specialty decision status in medical students at Imam Muhammad Ibn Saud Islamic University in 2016

As illustrated in Figure 1, 24% of the total respondents reported having chosen a specialty. The fifth year had the highest percentage of students who chose a specialty (40%). The frequency of students who reported having excluded undesired specialties increased progressively until peaking in the third year (33%). A similar trend in narrowing down choices was also observed, but with its peak occurring in the fourth year (63%). Negative responses progressively decreased with each academic year, despite a spike in response occurring in fourth-year students. This might be explained by the relatively smaller sample of students from the fourth year. Half the respondents reported not having a specific plan to acquire a spot in a residency program of their desired specialty, opting to focus currently on their studies. Figure 2 illustrates how students who were less conclusive in their choice of specialty were more likely to focus on current studies. A highly significant two-tailed correlation was found between having chosen the desired specialty and having a specific plan to reach that specialty (p= 0.000001).

Responses about behavior are presented in Table 4. 78% of students reported participating in extracurricular activities. Cross tabulation with having a plan to reach a desired specialty reveals that students participated in extracurricular activities for strengthening their CV in equal percentages among those who had a plan and those who didn’t. Students who did not have a plan to reach a specialty were more likely to not participate in extracurricular activities as compared to those who did have a plan (29% as compared to 11%). Moreover, students who reported having a plan had higher percentages of participating in activities that related to their desired specialty as compared to those who didn’t have a plan (19% compared to 8%). A highly significant correlation was found between reporting having a plan to reach a desired specialty and students’ perceived knowledge of the materials covered on license exams (p<0.001). Sixty-one percent reported having taken active measures to improve their English, and 55% reported having taken active measures to improve their research skills.

**Table 4:** Responses in the behavior section

	Responses		%
Have you chosen a desired specialty?	Yes		24.4%
	Few possibilities		45.6%
	Excluded some		13.3%
	No, it's too early		14.4%
	No		2.3%
Do you have a plan to reach your desired specialty	Yes		41.1%
	No		58.9%
Do you participate in extracurricular activities	Yes		78.4%
		Only ones related to my desired specialty	12.5%
		Yes, they strengthen my C.V	52.3%
		Yes, because I enjoy it	13.6%
	No		21.6%
Have you taken active measures to improve your language	Yes		61.4%
	No		38.6%
Have you taken active measures to improve your research skills	Yes		55.4%
	No		44.6%
Do you use recommended textbooks	Yes, as a main source		19.6%
	Sometimes		32.5%
	Only when needed		28.3%
	Rarely		17.4%
	Never		2.2%

## Discussion

Views toward the PBL system

Students have positive ideas regarding the PBL systems and the relevance of undergraduate studies to future practice. The positive outlook regarding the PBL system found in our study confirms the results of a previous study that showed both tutors and students agreeing on how the curriculum-selected contents were well adapted to the PBL [9].

Perceptions of own weaknesses

Students who stated they have poor knowledge of the type of material covered in license exams had higher chances of reporting having good knowledge about their own weaknesses. These findings suggest that those students might not be fully aware of what is expected of them. Having an annual session from a representative of the Saudi Commission of Health Specialties (SCHS) might help clear up any uncertainties that students seem to be having. 

School preparations

An interesting pattern seems to take place regarding students’ views of whether medical schools prepare them well for the license exams. We found that as students progressed from the first to the fifth year of medical school, their views seemed to change from negative to positive. First-year students reported feeling that their schools are not preparing them sufficiently but this tends to resolve as students gain more clinical experience towards their later years of studies.

Choice of future specialty

Our findings showed that the majority of students have not developed a strong decision and need more time to choose their desired specialty, which supports the findings reached by previous studies [10-12]. Insignificant changes in the numbers are present but those might have arisen from the detailed design of the questionnaire. Our results contradict those of other studies that showed higher percentages of specialty choice among students [13]. Mentorship programs can help connect students to faculty members in a way that supports their ability in making decisions regarding their future.

Extracurricular activities

Students who reported having a plan to reach the specialty of their desire tended to participate in activities related to those specialties. On the other hand, students who did not report having made a conclusive decision tended to participate in different types of activities with the main purpose of strengthening their CV. Extracurricular activities should be oriented toward students' interests to avoid the misuse of them as CV fillers.

Limitations

Due to the limited timeframe allocated to the study, we were unable to expand the sample in a way that allowed the inclusion of female students in the study. Additionally, we were unable to collect a sizeable amount of responses from fourth-year students.

## Conclusions

This study highlights the significant lack of knowledge of the medical students of the types of material that should be covered to pass the Saudi medical licensing exams. On the other hand, they have an adequate level of awareness and acknowledgment of their own weaknesses to work on improving themselves. Moreover, medical students show a positive attitude toward PBL, which fills the gaps in the system to connect and relates the undergraduate studies to clinical practice. The delay in perceiving the students’ own interests to focus on may lead to finding themselves unprepared at practicing clinical skills. However, more studies are mandatory to explore the reasons behind medical students’ lack of knowledge and the factors involved in choosing their specialties.
